# Elevated Incidence of Polyp Formation in APC^Min/+^Msh2^−/−^ Mice Is Independent of Nitric Oxide-Induced DNA Mutations

**DOI:** 10.1371/journal.pone.0065204

**Published:** 2013-05-31

**Authors:** Antoaneta Belcheva, Blerta Green, Ashley Weiss, Catherine Streutker, Alberto Martin

**Affiliations:** 1 Department of Immunology, University of Toronto, Toronto, Ontario, Canada; 2 Department of Laboratory Medicine, St. Michael’s Hospital, Toronto, Ontario, Canada; University of Pittsburgh, United States of America

## Abstract

Gut microbiota has been linked to a number of human diseases including colon cancer. However, the mechanism through which gut bacteria influence colon cancer development and progression remains unclear. Perturbation of the homeostasis between the host immune system and microbiota leads to inflammation and activation of macrophages which produce large amounts of nitric oxide that acts as a genotoxic effector molecule to suppress bacterial growth. However, nitric oxide also has genotoxic effects to host cells by producing mutations that can predispose to colon cancer development. The major DNA lesions caused by nitric oxide are 8oxoG and deamination of deoxycytosine bases. Cellular glycosylases that belong to the base excision repair pathway have been demonstrated to repair these mutations. Recent evidence suggests that the mismatch repair pathway (MMR) might also repair nitric oxide-induced DNA damage. Since deficiency in MMR predisposes to colon cancer, we hypothesized that MMR-deficient colon epithelial cells are incapable of repairing nitric-oxide induced genetic lesions that can promote colon cancer. Indeed, we found that the MMR pathway repairs nitric oxide-induced DNA mutations in cell lines. To test whether nitric oxide promotes colon cancer, we genetically ablated the inducible nitric oxide synthase (iNOS) or inhibited iNOS activity in the APC^Min/+^Msh2^−/−^ mouse model of colon cancer. However, despite the fact that nitric oxide production was strongly reduced in the colon using both approaches, colon cancer incidence was not affected. These data show that nitric oxide and iNOS do not promote colon cancer in APC^Min/+^Msh2^−/−^ mice.

## Introduction

The mismatch repair (MMR) pathway maintains genomic stability, and loss of MMR function is associated with hereditary nonpolyposis colorectal cancer (HNPCC, i.e. Lynch Syndrome) in mice and humans [1], [2]. However, the mechanistic linkage between MMR-deficiency and the etiology of CRC remains elusive. The MMR pathway is an evolutionarily conserved DNA repair pathway that recognizes and repairs DNA mismatches that are either generated by incorporation of the wrong nucleotide during DNA replication or through other genetic events that lead to mispaired DNA [3]. Failure to repair mispaired DNA due to deficiencies in Msh2 or Msh6 (i.e. proteins essential for MMR) leads to accumulation of mutations throughout the genome. Consequently, this may lead to loss of function in tumor suppressor genes or activation of oncogenes [4]. Indeed, MMR-deficiency results in elevated rates of mutations in essential genes that govern normal colonic epithelial growth [1]. Patients with germ line mutations in MMR genes are prone to develop CRC in 80% of the cases [5], [6], [7]. However, based on the role of MMR in suppressing mutations generated by replication errors in all cell types, it remains a mystery as to why MMR-deficiency predisposes to CRC more so than to other cancers.

The gut is a natural habitat for a large number of bacterial species. Major functions of the gut microflora include metabolic activities that result in salvage of energy and absorbable nutrients, and protection of the host against invasion by pathogenic microbes [8]. However, in some cases this mutually beneficial relationship goes awry, and the gut flora instead contribute to the development of pathological disorders, including CRC and inflammatory bowel diseases [8], [9], [10], [11]. The mechanism through which gut flora mediate CRC development is still poorly understood. The immune system plays an important role in controlling the homeostasis between the gut flora and host, one example being the production of nitric oxide by inflammatory monocytes [12]. Importantly, inflammatory processes such as gastritis and colitis that involve constant macrophage activation are considered as high risk factors for CRC [13], [14]. In the inflamed tissue, macrophages are activated by endogenous IFN-γ or bacterial products such as LPS which leads to expression of the inducible nitric oxide synthase (iNOS), an enzyme that generates high amounts of nitric oxide [15],[16],[17],[18]. As part of the host defense, high concentrations of nitric oxide have cytopathic effects on intestinal flora but also cause collateral DNA damage in host tissue. By reacting with oxygen or superoxide radical, nitric oxide generates highly reactive species such as nitrous anhydride (N_2_O_3_) and peroxynitrite (ONOO^−^). These species cause direct and indirect DNA damage [19], [20]. The most common DNA alteration is accumulation of 8-oxodG as well as deamination of deoxycytosine nucleotides producing deoxyuracil:deoxyguanine (dU:dG) mispairs [21], [22]. Enzymes such as uracil DNA glycosylase and Ogg1 have been shown to be critical in repairing oxidative DNA damage [23], [24]. However, we and others have provided evidence that the MMR pathway also repairs dU:dG mispairs that are caused by the enzyme activation-induced cytidine deaminase [25], [26], [27], an enzyme that is critical for an effective humoral response in immunity. Thus, nitric oxide may produce a substrate for the MMR pathway. Deficiency in MMR in the epithelial tissue lining the gut may lead to elevated mutation rates in these tissues and a consequent increase in the risk of developing CRC. In this regard, it is important to understand whether the MMR pathway plays a role in the repair of nitric oxide-induced-DNA damage and if so, whether the consequent accumulation of mutations has the capacity to enhance CRC development in MMR-deficient hosts. The role of nitric oxide in colon carcinogenesis is unclear mainly due to the complexity of its physiological effects under normal and pathological conditions [28]. Furthermore, the mechanism through which iNOS and its product nitric oxide influence CRC development in mice harboring mutations in the adenoma polyposis coli gene (APC^Min/+^) is controversial [29], [30], [31] prompting us to examine its role specifically in the context of APC^Min/+^Msh2^−/−^ mouse model of CRC.

The present study provides evidence that the MMR pathway repairs mutations that are generated by nitric oxide. However, inhibiting iNOS activity or genetically ablating iNOS did not alter CRC development in APC^Min/+^Msh2^−/−^ mice indicating that nitric oxide does not significantly contribute to colon cancer etiology in this mouse model.

## Results

**Figure 1:**
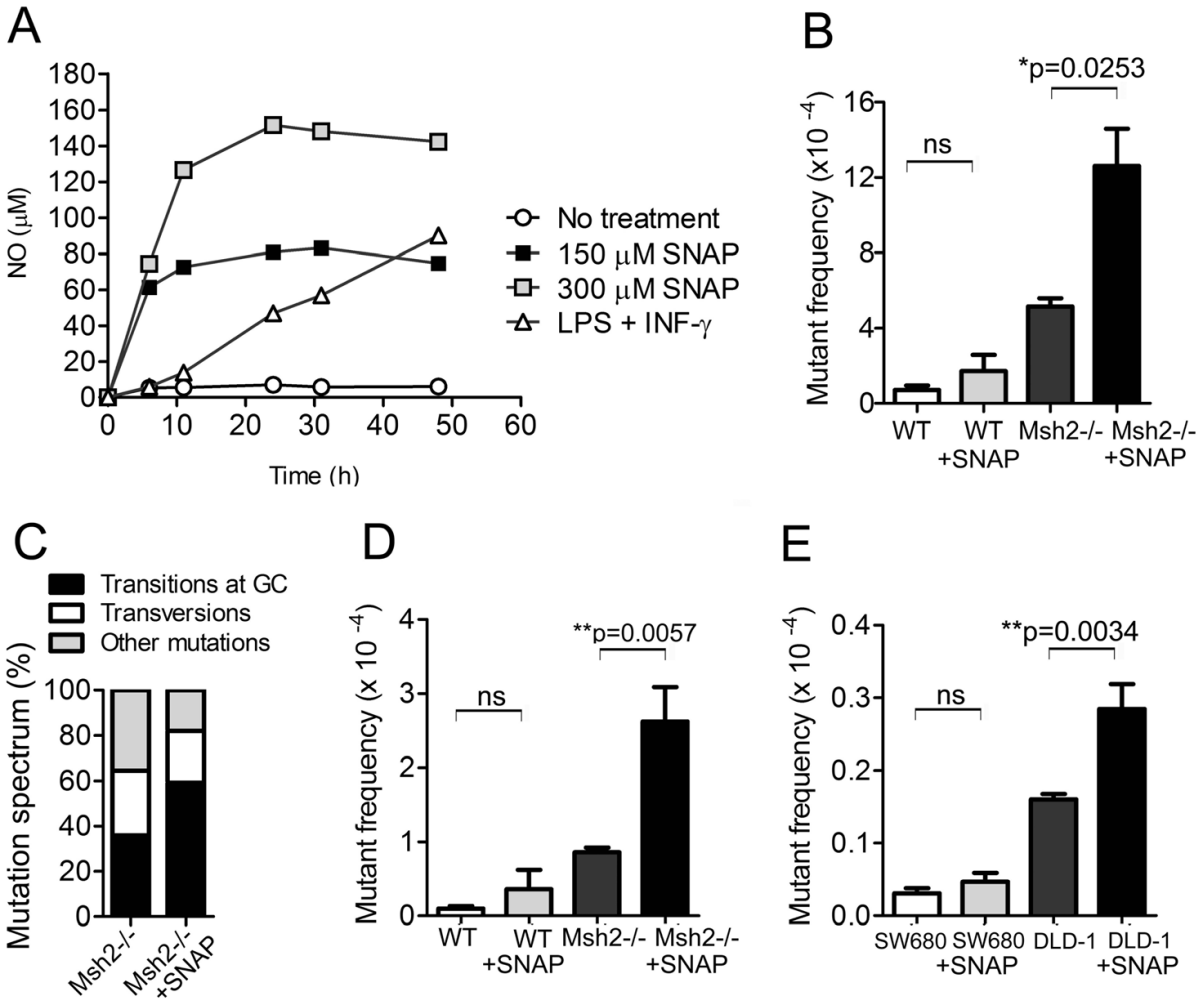
Nitric oxide induces DNA mutations that are repaired by the MMR pathway. (A) Measurements of the amounts of nitric oxide in culture medium generated by various concentrations of SNAP or by macrophages that were stimulated by LPS and IFN-γ. (B) The mutant frequency at the *lac*I gene in Msh2^+/−^ (WT, n = 3) and Msh2^−/−^ (n = 3) SNAP-treated or non-treated macrophages was measured by the Big Blue mutagenesis screen. Bone marrow-derived macrophages were cultured in presence of 150 µM SNAP for 20 hrs. Genomic DNA was isolated and the mutant frequency at *lac*I gene was calculated. (C) Mutation spectrum in SNAP-treated macrophages as determined by sequencing analysis. The C:G → T:A transitions are shown. The tranversion mutations include C:G → A:T, C:G → G:C, T:A → G:C and T:A → A:T substitutions. The insertions and deletions are presents as other mutations. (D) The mutant frequency at the *hprt* gene in Msh2^+/−^ (WT, n = 8) and Msh2^−/−^ (n = 8) SNAP-treated or non-treated Pre-B cells. (E) The mutant frequency at the *hprt* gene in SW680 (n = 8) and DLD-1 (n = 8) SNAP-treated or non-treated human colorectal cancer cell lines.

Nitric oxide has been shown to play a pivotal role in cellular transformation [32], [33]. One of the mechanisms through which nitric oxide elicits these effects is by causing extensive DNA damage throughout the genome leading to gain-of-function and/or loss-of-function mutations in tumor suppressor or proto-oncogenes [32], [33]. Although DNA glycosylases are involved in the repair of the mutations caused by nitric oxide, it is currently not clear whether the MMR pathway also plays a role in the repair process. Hence, we tested whether the MMR pathway repairs mutations induced by nitric oxide in various cell types. For this purpose we used S-nitro-N-acetyl-D L-penicillamine (SNAP) as a nitric oxide donor since SNAP releases substantial amounts of nitric oxide when dissolved in culture medium [34]. First, we determined what concentration of SNAP generates an amount of nitric oxide that is similar to the physiological levels of nitric oxide produced by macrophages that are stimulated by IFN-γ and LPS (Figure 1A). We treated macrophages derived from wild type (Msh2^+/−^) and Msh2^−/−^ mice with different concentrations of SNAP and measured the amount of nitric oxide in the culture medium over time. These analyses showed that 150 µM of SNAP produced similar amount of nitric oxide to that produced by IFN-γ and LPS-activated macrophages for 48 hours, although with different kinetics.

To test whether the MMR pathway repairs mutations generated by reactive nitric oxide species (RNOS), we measured the mutant frequencies in Msh2^+/−^ and Msh2^−/−^ macrophages that were SNAP-treated or untreated using the *lacI* transgene reporter system (BigBlue®). This system has been widely used to measure mutant frequencies caused by environmental mutagens or mutations due to DNA repair defects [35]. We found that SNAP-treatment leads to a significant increase in mutant frequency specifically in Msh2^−/−^ macrophages, but not in Msh2^+/−^ macrophage controls (Figure 1B). We next examined the characteristics of mutations in the *lacI* gene in macrophages that were exposed to high amounts of nitric oxide. These mutations were compared to the mutation spectrum in WT and Msh2^−/−^ macrophages that were not treated with SNAP [36]. The background mutant frequency was increased ∼2-fold in SNAP treated Msh2^−/−^ macrophages (Figure 1B). This value correlated with a 24% increase in transition mutations at dC:dG base pairs (C:G→T:A transitions) (Figure 1C), (Table 1). This result indicates that the predominant SNAP-induced mutations were deoxycytidine deaminations.

**Table 1:** Characteristics of the mutations within the *lacI* gene in Msh2 WT and Msh2^−/−^ macrophages that were SNAP-treated or untreated.

	*Msh2^+/+^ (%)*	*Msh2^−/−^ (%)*	*Msh2 ^−/−^ SNAP (%)*
**Transitions**	**3 (42.8)**	**6 (42.9)**	**15 (68.2)**
**C:G→T:A all**	3 (42.8)	5 (35.7)	13 (59.1)
**CpG sites**	3 (42.8)	3 (21.4)	8 (36.4)
**Non CpG sites**	0 (0)	2 (14.3)	5 (22.7)
**TA→C:G**	0 (0)	1 (7.1)	2 (9.1)
**Transversions**	**3 (42.8)**	**4 (28.6)**	**5 (22.7)**
**C:G→T:A**	2 (28.6)	1 (7.1)	1 (4.5)
**C:G→G:C**	1 (14.3)	0 (0)	0 (0)
**T:A→G:C**	0 (0)	1 (7.1)	3 (13.6)
**T:A→A:T**	0 (0)	2 (14.3)	1 (4.5)
**Insertions/deletions**	**1 (14.3)**	**4 (28.6)**	**2 (9.1)**
**Insertions**	0 (0)	2 (14.3)	0 (0)
**Deletions**	1 (14.3)	2 (14.3)	2 (9.1)
**Total mutations**	**7 (100)**	**14 (100)**	**22 (100)**

We further investigated whether the MMR pathway repairs nitric oxide-induced DNA mutations in other types of cells. For this analysis, we used MMR proficient (15–63) and deficient pre-B cell lines (8–58: Msh2^−/−^), and MMR proficient (SW680) and deficient (DLD-1; Msh6-deficient) human colon cancer cell lines. Using the HPRT mutation assay, we showed that SNAP-treatment leads to a ∼2-fold increase in the mutant frequency in Msh2^−/−^ pre-B cells (Figure 1D) and in the MMR-deficient human colon cancer cell line DLD-1 (Figure 1E), but not in their respective controls. Taken together these data confirm the importance of the MMR pathway as a major DNA repair pathway of nitric-oxide induced mutations.

**Figure 2:**
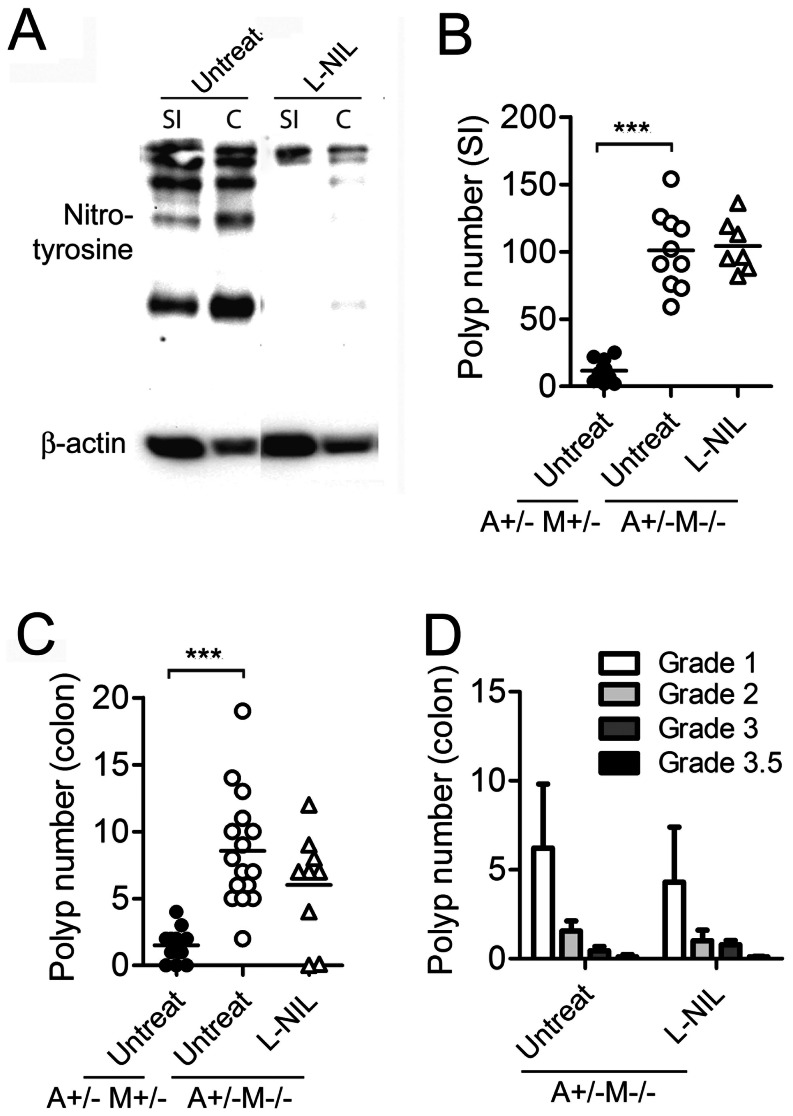
L-NIL treatment does not reduce polyp numbers in APC^Min/+^MSH2^−/−^ mice. (A) iNOS activity in the small intestine (SI) and colon (C). Anti-nitrotyrosine antibody was used in a western blot to detect nitrated proteins in epithelial cells derived from the small intestine and colon of APC^Min/+^MSH2^−/−^ mice that were untreated, or N^6^-(1-immunoethyl)-L-lysine dihydrochloride (L-NIL)-treated. (B) Polyp count in small intestine of APC^Min/+^MSH2^+/−^ (A^+/−^M^+/−^) mice and APC^Min/+^MSH2^−/−^ (A^+/−^M^−/−^) mice untreated or L-NIL treated. (C) Polyp count in colon of APC^Min/+^MSH2^+/−^ (A^+/−^M^+/−^) mice and APC^Min/+^MSH2^−/−^ (A^+/−^M^−/−^) mice untreated or L-NIL treated. (D) Pathology scores for the polyps observed in the small intestines and colon of A^+/−^M^+/−^ and A^+/−^M^−/−^ mice untreated or L-NIL-treated.

The role of iNOS in different stages of colon carcinogenesis has been highlighted in numerous studies [28], [37], [38] and highly selective iNOS inhibitors were developed and tested for chemoprevention of colon cancer [39], [40], [41]. However, the role of iNOS specifically in the APC^Min/+^ (multiple intestinal neoplasia) model of CRC remains controversial owing to the different results obtained [29], [31]. In the gut, nitric oxide is generated in response to commensal flora by inflammatory monocytes and B cells [42] and our results demonstrate that the MMR pathway repairs nitric oxide-induced mutations. In light of these observations, we hypothesized that commensal flora would induce nitric oxide production by inflammatory lymphocytes/monocytes that cause DNA lesions in epithelial tissue that are normally repaired by MMR pathway. Hence, Msh2-deficiency might lead to accumulation of RNOS-induced genetic lesions which may result in enhanced CRC development. To test whether RNOS stimulate CRC in APC^Min/+^MSH2^−/−^ mice, we administered the potent iNOS inhibitor L-NIL in the drinking water continuously for 6 weeks. We first assessed whether L-NIL effectively suppressed iNOS activity *in vivo.* Since nitric oxide can react with tyrosine residues expressed on proteins in gut epithelial cells, we used an anti-nitrotyrosine antibody to determine whether the levels of nitrated proteins were reduced by L-NIL treatment. Epithelial cell lysates were prepared from the small intestine and colon of L-NIL-treated mice, and the presence of nitrated proteins was visualized by western blot. The results revealed that L-NIL treatment was sufficient to suppress iNOS activity as we detected fewer nitrated proteins in extracts from small intestine and colon epithelial cells derived from L-NIL-treated mice (Figure 2A).

We next assessed whether RNOS stimulates polyp number in APC^Min/+^MSH2^−/−^ mice. Compared to APC^Min/+^Msh2^+/−^ mice, APC^Min/+^Msh2^−/−^ mice had significantly enhanced polyp formation in the small intestine (Figure 2B) and colon (Figure 2C) at 6 weeks of age. However, L-NIL treatment had no effect on polyp number in APC^Min/+^MSH2^−/−^ mice (Figure 2B,C). Furthermore, we graded the polyps according to established histopathological criteria (see Materials and Methods) and this analysis showed that L-NIL treatment did not alter the severity of the disease (Figure 2D).

**Figure 3:**
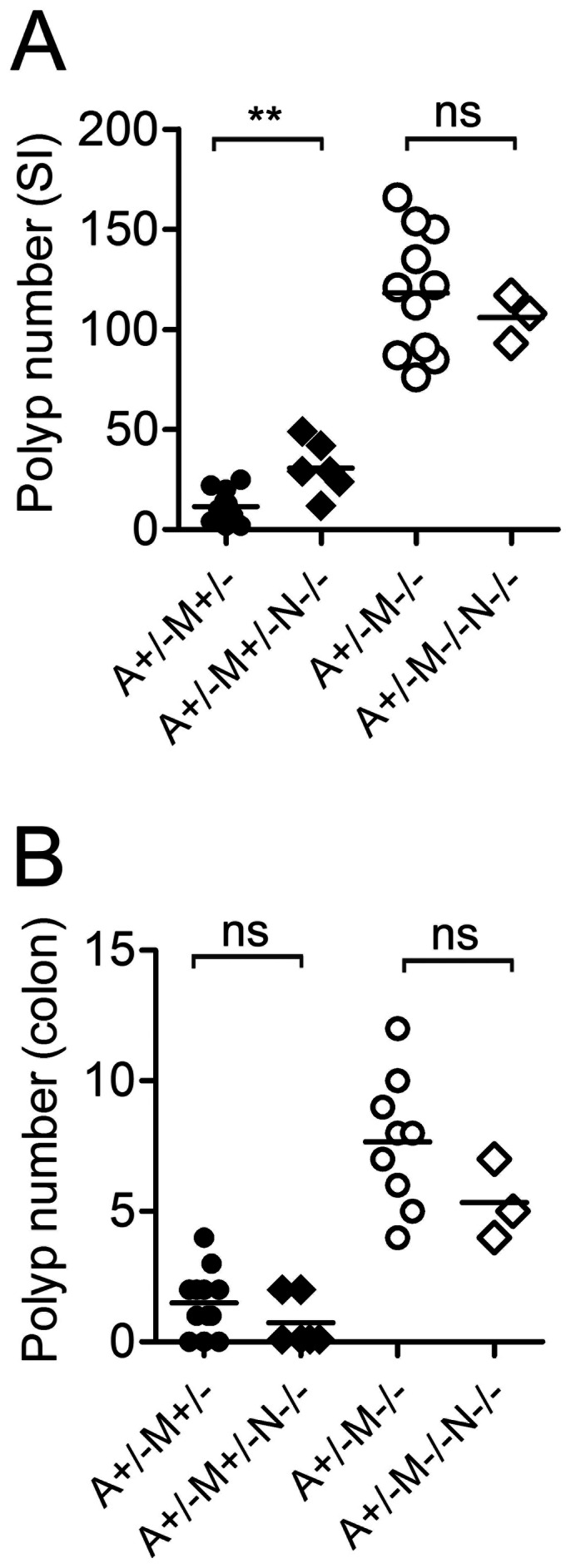
iNOS-deficiency does not impact polyp number in APC^Min/+^MSH2 ^−/−^ **mice.** Polyp count in the small intestine (A) or in the colon (B) of APC^Min/+^MSH2^+/−^ (A^+/−^M^+/−^) mice and APC^Min/+^MSH2^−/−^ (A^+/−^M^−/−^) mice that were iNOS proficient (N^+/−^) or iNOS deficient (N^−/−^).

Because of the possibility that L-NIL-treatment may affect other cellular processes in addition to nitric oxide production, we examined polyp number in APC^Min/+^MSH2^+/−^ and APC^Min/+^MSH2^−/−^ mice in the iNOS^−/−^ background. We found that the polyp number was enhanced in the small intestine, but not in the colon, of APC^Min/+^MSH2^+/−^ iNOS^−/−^ mice compared to APC^Min/+^MSH2^+/−^ controls (Figure 3A, B). However, genetic ablation of the iNOS gene had no effect on polyp number in both the small intestine and colon of APC^Min/+^MSH2^−/−^ iNOS^−/−^ mice compared to their iNOS–sufficient littermates (Figure 3A, B). Taken together, these results show that although nitric oxide increases the background mutation frequency in MMR-deficient cell lines, nitric oxide does not enhance polyp development in the APC^Min/+^MSH2^−/−^ genetic model of CRC.

## Discussion

Nitric oxide has the capacity to induce extensive DNA damage [21], [22], [33] and to inhibit DNA repair processes in exposed cells [24], [43], [44]. Oxidative DNA damage, like that caused by nitric oxide, is typically repaired by DNA glycosylases such as OGG1, UNG, TDG, SMUG1, and MBD4 [23], [24], [45]. Since MMR repairs many types of mutations, many of which overlap with the DNA repair specificities of these DNA glycosylases, we hypothesized that MMR might suppress nitric oxide DNA lesions. This mechanism might provide an explanation for why MMR-deficiency is associated with CRC etiology. However, the role of the MMR pathway in the repair of nitric oxide-induced mutations has never been examined before. We demonstrated that nitric oxide induces significantly higher mutation frequency only in cells that are deficient in Msh2 or Msh6. In contrast, nitric oxide had no effect on the mutation frequency of normal controls (Figure 1) supporting the notion that MMR actively repairs lesions caused by nitric oxide.

Deficiencies in MMR have long been linked to colon cancer, and considering that somatic mutations are generally a prerequisite for cancer, it is puzzling that MMR deficiency occurs more frequently among patients with colon cancer than for other cancers. In the gut, nitric oxide is produced by both gut microbiota [46] and cells of the immune system [42], [47]. In light of the above findings that MMR repairs mutations arising from nitric oxide, we hypothesized that nitric oxide produced by inflammatory lymphocytes/monocytes in response to gut microbiota might be an important etiologic factor in colon epithelial cell carcinogenesis. However, our results show that nitric oxide does not significantly impact polyp formation in APC^Min/+^MSH2^−/−^ mice. Previous work on the role of iNOS and its product nitric oxide on CRC development in APC^Min/+^ mice is controversial. The absence of iNOS reduced tumor formation in APC^Min/+^ mice [29], [30]. However, another study suggested a pro-neoplastic role for nitric oxide in the same mouse model [31]. These contradictory results have been suggested to be due to differences in mouse diets and more specifically to the availability of dietary arginine [30] as well as the different sensitivity of the cancer cells to nitric oxide [48]. Indeed, Yerushalmi et al. demonstrated that the impact of iNOS-deficiency on polyp growth was different in the small intestine and colon, and supplemental arginine (0.2%–2%) had variable effects. [30]. In our study, mice were fed with Teklad global 18% protein diet that is similar to the AIN-93G diet in terms of dietary arginine and this may explain the lack of effect of iNOS deficiency on polyp incidence. However, in the experiments with L-NIL, we detected substantial reduction of the nitrated proteins that correlates with low production of nitric oxide in the gut of APC^Min/+^MSH2^−/−^ mice and this had no effect on the intestinal polyposis. Interestingly, a number of iNOS inhibitors such as phenyl*bis*isothiourea (PBIT), NILT (structural analog of L-NIL) and aminoguanidine hydrochloride (AG) have been successfully used to significantly reduce aberrant crypt foci formation in AOM-induced CRC cancer [49], [50] and NILT alone has been shown to suppress inflammation [39]. These differing effects of iNOS on colon cancer development are likely due to the complexity of interactions between the host immune system, host genetics, and gut microbiota. Undoubtedly, under inflammatory conditions, nitric oxide is released in substantial amounts that can cause DNA damage as well as shape the entire microbiota giving growth advantage to nitrate-respiration proficient Enterobacteriaceae, specifically *E. coli* and lead to dysbiosis [51]. Outgrowth of *E. coli* has been shown to induce DNA-damage in the colon epithelial cells and is linked to colon cancer development [10], [11]. Despite our finding that MMR can repair mutations generated by NO *in vitro*, it is very likely that the majority of mutations that arise in MMR-deficient mice are due to replication errors. Hence, the strong mutator phenotype in APC^Min/+^MSH2^−/−^ mice might obscure a role for NO in CRC development. Although there was a small increase in polyp number in the small intestine in iNOS-deficient APC^Min/+^MSH2^+/−^ mice relative to APC^Min/+^MSH2^+/−^ mice, this was not the case in the colon (Fig. 3) suggesting that NO has at best a minor role in CRC induction in MMR-proficient animal models

In conclusion, this study has led to two important observations. First, we demonstrated that the MMR pathway repairs mutations generated by nitric oxide. Second, nitric oxide that is normally produced in the gut is not an etiological factor that contributes to accelerated polyp development in the APC^Min/+^MSH2^−/−^ mice. Indeed, it has been shown that nitric oxide is produced in greater concentrations in patients with ulcerative colitis [13], [14] suggesting that nitric oxide may play an important role in the process of epithelial cell transformation in colitis-associated CRC.

## Materials and Methods

### Mice

Msh2^+/−^ mice were kindly provided by Dr. Tak W. Mak (Ontario Cancer Institute). Male C57BL/6J-APC^Min/+^ was obtained from The Jackson Laboratory (Bar Harbor, ME). Male APC^Min/+^ mice were crossed with Msh2^+/−^ female mice to generate APC^Min/+^Msh2^+/−^ male mice. APC^Min/+^Msh2^+/−^ males were further bred with Msh2^+/−^ females to generate APC^Min/+^Msh2^+/−^ and APC^Min/+^Msh2^−/−^ offspring. The genotyping for APC^Min/+^ was carried out by PCR method described previously [52]. The genotyping for Msh2 was done as described previously [53]. For mutation analysis we used Big Blue C57BL/6 hemizygous male mice that were purchased from Stratagene. Msh2^−/−^ mice were crossed with Big Blue (BB^+^) to generate BB^+^Msh2^+/−^ and BB^+^Msh2^−/−^ offspring. The mice that carry the shuttle vector (BB+) were identified by PCR as we have already described [36]. The iNOS^−/−^ mice were obtained from Dr. J Gommerman (Toronto). The animals were raised under specific pathogen-free conditions. The animals were fed with Teklad Global 18% protein rodent diet (Harlan, WI), that contains 1% dietary arginine. At the age of 6 weeks the mice were sacrificed by cervical dislocation.

### Cell culture

Bone marrow (BM) cells were harvested from ∼3-month old WT and Msh2^−/−^ mice carrying the *lacI* transgene and differentiated into macrophages by culturing the cells in the presence of GM-CSF as described previously [54]. Briefly, the cells were harvested from the femur and tibia by flushing the bones with a syringe carrying PBS. The red blood cells were lysed with RBC lysis buffer (Sigma). The BM cells where differentiated into macrophages by culturing the BM precursors for 6 days in RPMI 1640 (10% FCS, 10% L-glutamine, 10 penicillin/streptomycin) in the presence of 10 ng/ml recombinant murine (rm)GM-CSF (PeproTech). About 8 to 10 million cells were cultured in each dish. On Days 3 and 5 of the culturing period, 70% of the culture supernatant was removed, which contained non-adherent cells, and replaced with fresh media containing rmGM-CSF at 10 ng/ml. On Day 6, the loosely adherent and non-adherent cells (representing granulocytes and dendritic cells) were removed by vigorous washing twice with PBS. The remaining adherent macrophage population was harvested by incubating the cells for 5 min in PBS containing 10 mM EDTA at 37°C followed by gentle scraping with rubber policemen (Starsted). The harvested macrophages were used for DNA isolation and mutation analysis. The SW680 (MMR proficient) and DLD-1 (MMR deficient, MSH 6^−/−^) human colon cancer cell lines were cultured and passaged in Dulbecco’s modified Eagle’s medium (DMEM) supplemented with penicillin (100 U/ml), and streptomycin (0.1 mg/ml; Sigma) 10% fetal calf serum (Gibco). Abelson pre-B cell lines 15–63 (Msh2^+/−^) and 8–58 (Msh2^−/−^) were maintained in RPMI medium (Invitrogen) with 10% bovine calf serum (HyClone), penicillin (100 U/ml), and streptomycin (0.1 mg/ml; Sigma).

### Measurements of the nitric oxide levels in cell culture media

Macrophages were activated with 100 U/ml IFN-γ and LPS 20 ng/mL (Sigma). S-nitro-N-acetyl-D, L-penicillamine (SNAP) was purchased from Cayman chemicals and was added to the cell culture medium. Samples were removed at indicated time points and the concentration of nitric oxide was measured using the Griess reagent system (Promega) as described in the manufacturing protocol.

### Mutation analysis

Tissue preparation and transgenic λ phage rescue was carried out as described previously [35]. Phage was packaged with the Stratagene Transpack Packaging extract (Sigma-Aldrich). The Big Blue mutation screen was performed as we have described before [36]. The dilution assay was performed on 10-cm plates with SCS-8 bacterial lawn and the next day the virus was plated on 15-cm plates in presence of X-gal. *LacI* mutant frequency was calculated by determining the ratio of mutant (blue) plaques to non-mutant (colorless) plaques. To ensure accurate mutant frequencies, at least 300 000 plaque forming units (pfu) were plated per tissue per animal. For the *hprt* mutation analysis, we first selected cells with wild type *hprt* gene. The cells were cultured in hypoxanthine-aminopterin-thymidine (HAT) media (Sigma) for 3 weeks and passaged every 2–3 days. The cells then were SNAP- treated or non-treated and plated in 96 well plates at concentration 1×10^4^ in presence of 6-Thioguanine (6-TG) at a final concentration 1.5–2.5 µg/mL. The plates were then incubated for 2 weeks at 37°C and the surviving clones were counted.

### Sequencing

The blue plaques from the Big Blue mutation screen were used to PCR amplify the *lacI* region and *LacZ* promoter (1338 bp segment) with primers previously reported (-55D: 5′-GTA CCC GAC ACC ATC GAA TG3′ and 1283U: 5′-GAG TCA CGA CGT TGT A 3′) [55]. The PCR products were purified by a MultiScreen PCR_96_ Filter Plate (Millipore) and sent to Macrogen (Korea) for sequencing using primers -55D (details found above) and 1232U: 5′GAA TCC GTA ATC ATG GTC ATA 3′ [55]. Alignment and analysis of unique mutations was done using SeqMan.

### Histopathology

The entire small intestine and colon were removed and washed in cold PBS. Then the intestines were opened longitudinally and the polyps were evaluated under stereoscopic microscope. The intestines were prepared by the Swiss roll method and the polyps were evaluated from H&E stained sections. Displasia/neoplasia scores used to estimate the severity of the cellular changes were as described previously [56], [57].

### L-NIL treatment

Pregnant mice were put on _L_-N^6^-(1-iminoethyl)-lysine (L-NIL, Cayman chemical) treatment that was added in their drinking water at final concentration 500µg/ml. The L-NIL containing water was replaced every 4 days. At 6 weeks of age the mice were sacrificed and the tumor load was estimated as we described above.

### Western Immunoblot for detection of nitrated proteins

Western blot using the anti-nitrotyrosine antibody (Millipore) was carried out on epithelial cells from small intestine and colon of untreated, antibiotics-treated or L-NIL treated mice. Membranes were then blotted with HRP-conjugated anti-rabbit IgG secondary antibody and the nitrated proteins were detected by chemiluminescence.

### Statistical analysis

All analyses were performed using GraphPad Prism version 5.0 in which we performed 2-tailed student *t* test.

### Ethics Statement

All animal study protocols were approved by the University of Toronto’s Division of Comparative Medicine Committee.
